# NADPH oxidase 4 is protective and not fibrogenic in intestinal inflammation

**DOI:** 10.1016/j.redox.2020.101752

**Published:** 2020-10-07

**Authors:** Emily Stenke, Gabriella Aviello, Ashish Singh, Sean Martin, Des Winter, Brian Sweeney, Michael McDermott, Billy Bourke, Seamus Hussey, Ulla G. Knaus

**Affiliations:** a Conway Institute, School of Medicine, University College Dublin, Dublin, Ireland; b St. Vincent's University Hospital, Dublin, Ireland; c National Children's Research Centre, Children's Health Ireland, Dublin, Ireland; d RCSI University of Medicine and Health Sciences, Dublin, Ireland

**Keywords:** Intestinal inflammation, Fibrosis, Crohn's disease, NADPH oxidase, NOX4

## Abstract

Dysregulated redox signaling and oxidative injury are associated with inflammatory processes and fibrosis. H_2_O_2_ generation by NOX4 has been suggested as a key driver in the development of fibrosis and a small molecule drug is under evaluation in clinical trials for idiopathic pulmonary fibrosis and primary biliary cholangitis. Fibrosis is a common complication in Crohn's disease (CD) leading to stricture formation in 35–40% of patients, who require surgical interventions in the absence of therapeutic options. Here we assess NOX4 expression in CD patients with inflammatory or stricturing disease and examine whether loss of NOX4 is beneficial in acute and fibrotic intestinal disease. *NOX4* was upregulated in inflamed mucosal tissue of CD and ulcerative colitis (UC) patients, in CD ileal strictures, and in mice with intestinal inflammation. Nox4 deficiency in mice promoted pathogen colonization and exacerbated tissue injury in acute bacterial and chemical colitis. In contrast, in two chronic injury models aberrant tissue remodeling and fibrosis-related gene expression did not differ substantially between *Nox4*^*−/−*^ mice and wildtype mice, suggesting that Nox4 is dispensable in TGF-β1-driven intestinal fibrogenesis. While animal models do not recapitulate all the hallmarks of CD fibrosis, the tissue-protective role of Nox4 warrants a cautious approach to pharmacological inhibitors.

## Highlights

• *NOX4* is elevated in inflammatory bowel disease strictures and inflamed biopsies.
• Murine intestinal fibrogenesis is not dependent on Nox4.
• Nox4 is protective in acute murine colitis.

## Graphical abstract

**Figure:**
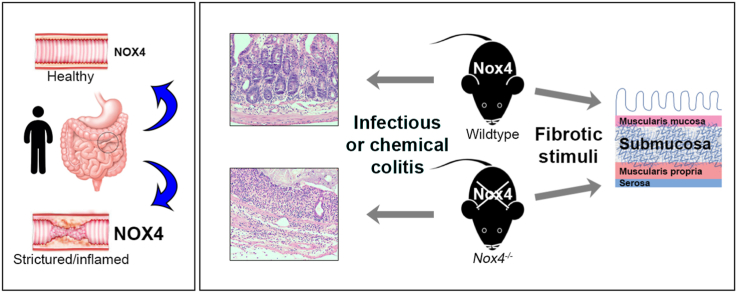

## 1 Introduction

Inflammatory bowel disease (IBD), characterized by chronic inflammation of the gastrointestinal tract, includes Crohn's disease (CD) and ulcerative colitis (UC). After a continuous increase of IBD incidence and prevalence in developed countries during the last decades, IBD incidence appears to have stabilized in North America and Europe but is now rising in newly industrialized countries [1]. CD can affect any section of the gastrointestinal tract, commonly the terminal ileum and/or colon, and is characterized by discontinuous transmural inflammation featuring granulomas, whereas UC shows continuous mucosal inflammation confined to the colon. Intestinal fibrosis is a common long-term complication of both CD and UC, but affects CD patients more severely due to the progression to strictures which lead to ileal narrowing and blockage. Pediatric-onset CD is associated with higher rates of multiple strictures and earlier surgeries [2], with a high incidence of surgical complications including anastomotic disease recurrence and short bowel syndrome.

Fibrosis evolves from essential tissue regeneration processes where activated mesenchymal cells produce extracellular matrix (ECM) for wound repair. However, in pathological conditions persistent stimulation can create feed-forward loops leading to excessive ECM deposition. Transforming growth factor-β1 (TGF-β1) together with insulin-like growth factor-1 (IGF-1) act as key regulators of intestinal fibrogenesis, and both bioactive factors are upregulated in IBD patients [[3], [4], [5]]. Multiple signaling pathways including the canonical SMAD pathway and non-canonical MAP kinase, PI-3 kinase, and Rho GTPase pathways are involved in promoting TGF-β′s profibrogenic activity. An additional hallmark of TGF-β signaling is its complex relationship with reactive oxygen species (ROS), redox signaling and cellular antioxidant systems [6,7]. TGF-β1 was shown to increase ROS generation by inducing NADPH oxidase expression and/or by disrupting mitochondrial complex III and IV activity. In addition, TGF-β1 induced suppression of antioxidant enzymes required for de novo glutathione biosynthesis, for hydrogen peroxide (H_2_O_2_) degradation or superoxide conversion has also been observed.

The NADPH oxidase NOX4, a constitutively active H_2_O_2_ generating enzyme, was not only upregulated by several TGF-β-initiated signaling cascades, but can also amplify and perpetuate the signal by activating latent TGF-β1 and further stimulating TGF-β1 expression [6]. Many profibrotic effects of TGF-β1 have been linked to NOX4 activity such as myofibroblast differentiation, epithelial apoptosis, and epithelial-mesenchymal transition (EMT) of various cell types. In lung fibroblasts obtained from idiopathic pulmonary fibrosis (IPF) patients NOX4 contributed to TGF-β1-dependent smooth muscle actin upregulation, resistance to apoptosis and ECM secretion [6]. Apoptosis resistance and senescence of myofibroblasts derived from IPF patients and aged mice have been connected to a redox imbalance, resulting in increased NOX4 and decreased NRF2 levels [8]. Additionally, the hyaluronic acid receptor CD44v6 was reported as a critical component of a positive feedback loop coupling CD44v6 to the TGF-β receptor 1 and NOX4, leading to sustained SMAD signaling and promoting fibrogenesis [9]. NOX4 was not only upregulated in myofibroblastic foci in IPF, but also in nonalcoholic steatohepatitis and in hepatitis C virus-induced fibrosis [[10], [11], [12]], and deleting the *Nox4* gene proved beneficial in several animal models of lung and liver fibrosis [[13], [14], [15], [16]].

Based on these observations TGF-β1-induced redox signaling, and in particular by NOX4, is considered a promising drug target and new therapeutic option for fibrotic diseases. The compounds GKT136901 and GKT831 (Setanaxib), while not specific for NOX1/4 or NADPH oxidases in general [17,18], seem to act as general reducing agents and have performed well in animal models of fibrosis. Setanaxib is currently under evaluation in phase I/II clinical trials for IPF and primary biliary cholangitis. It has been challenging to discover truly selective small molecule NOX4 inhibitors with some putative candidates in the early development phases (GLX7013114/Glucox [19], Fibronox). No treatments are currently available for intestinal fibrotic disease, and novel drugs targeting redox signaling and NOX4 could be exploited for use in CD therapy. Studies examining the relationship of TGF-β and NOX4 in the intestine are very limited. *NOX4* expression was upregulated in intestinal myofibroblasts derived from three patients with fibrostenotic CD [20], and similar to reports on myofibroblasts derived from other tissues, TGF-β-stimulated collagen production in a murine myofibroblast cell line required the Smad-Nox4 pathway [21]. Whether Nox4 is also a critical signal conduit and amplifier in TGF-β1-mediated intestinal fibrosis has not yet been addressed in vivo. To provide a basis for considering NOX4-directed therapeutic intervention in IBD, this study set out to understand the role of NOX4 in IBD patients and in murine models of colitis and fibrosis.

## 2 Materials and methods

**Patient data and sample collection.** Colonic (cecum) and terminal ileal endoscopic biopsies from pediatric patients subsequently diagnosed as IBD or non-IBD from the DOCHAS study (GEN/193/11) were immediately immersed in RNAlater® and then stored at −80 °C. Samples from surgical resection specimens (ileal strictures or control ileum) were taken as follows: 1–3 stricture specimens, depending on the length of the stricture (minimum of 1 cm between specimens) and one control specimen from a grossly normal area of ileum near the resection margin. Non-IBD control ileal tissue was obtained from the site of ileostomy formation or closure in patients with colorectal cancer. At each location, paired full thickness and mucosal samples were stored in formalin and in RNAlater®.

**Animals.** SAMP1/YitFc mice (009355), Akr/J mice (000648), *Nox4*^*−/−*^ mice (022996) and C57BL/6J mice (000664) were from JAX®. *Nox4*^*−/−*^ mice were backcrossed to C57BL/6J mice at least five times and bred separately for several generations. Wildtype and *Nox4*^*−/−*^ mice (F0) were crossed to obtain heterozygote F1 which were intercrossed to produce F2 generation. Breeding of homozygous F2 animals produced the F3 generation *Nox4*^*+/+*^ (referred to as WT) and *Nox4*^*−/−*^ mice. Mice from F0, F2 and F3 generation were used for experiments. Mice for experiments were assembled by mixing when possible, and cages underwent block randomization in acute colitis models. Animals were housed in a specific pathogen free facility and received *ad libitum* purified water and irradiated chow (Teklad Global 18% Protein, Envigo). Daily scoring was performed during experiments for welfare parameters, body weight and disease activity index (DAI) as previously described [22].

**DSS colitis.** Mice were pre-conditioned for 10 days with 3–4 mice/cage. In the acute model, 7–10 week old male and female mice received 6 days of 2.5% DSS followed by water only for 3 days. In the chronic model, 8–11 week old female mice received 6 days of 2.5% DSS followed by water for 8 days, then 6 days of 3% DSS followed by water for 10 days, and were culled on day 30. One 50% bedding change was performed on day 10 of the chronic model. DSS (MW 40 kDa, batch DB001-37, TdB Consultancy) was prepared fresh every 3 days.

**Adherent-invasive *E. coli* (AIEC) infection.** 8–11 week old female mice were pre-conditioned for 8 days with 3–4 mice/cage. A 50% litter change was performed on day −1, day 3 and day 10. Mice were treated with 3% DSS in water (or water only) for 4 days prior to bacterial infection (day 0). AIEC (NRG857c (serotype O83:H1), kindly provided by Brian Coombes, McMaster University, Canada) [23,24] were cultured for 12h in Luria Bertain (LB) broth (Sigma) at 200 rpm shaking; mice were inoculated by oral gavage with 2 × 10^9^ CFU in 200 μl PBS or with PBS only on days 0, 2, 7 and 12.

***C. rodentium* infection.** The DPS100 strain of *C. rodentium* (kindly provided by Bruce Vallance, University of British Columbia, Canada) [25] was cultured in LB broth at 37 °C with 200 rpm shaking for 4h. Female and male mice (6–10 weeks) were inoculated with 1 × 10^9^ CFU in 200 μl of PBS by oral gavage or with PBS only.

**Pathogen colonization.** Feces (AIEC) or cecal/colonic content (*C. rodentium*) were homogenized in PBS, and plated at serial dilutions onto LB agar containing 35 μg chloramphenicol, 100 μg/ml ampicillin (AIEC) or chloramphenicol alone (*C. rodentium*), and cultured at 37 °C for 24h for bacterial enumeration.

**Histopathology.** Formalin-fixed distal colon or cecal apex of individual mice and full thickness ileal tissue from patients were embedded in paraffin and 5 μm sections were stained with H&E or Masson's trichrome. Slides were digitally scanned at 20x magnification by Aperio ScanScope XT scanner (Leica Biosystems) and saved in ScanScope Virtual Slide (.svs) format. Images were taken using AperioImage Scope software (Leica Biosystems). Histological inflammation in murine colon and cecum was assessed by a researcher blinded to group allocation, using a scale from 0 to 4 for AIEC and *C. rodentium* models [26] and a scale from 0 to 10 for the DSS model [27]. Muscularis propria and submucosa thickness were measured in Masson trichrome-stained transverse sections. For each technical replicate two measurements were made, one each at the thickest and thinnest part. The mean of these two measurements was calculated for each technical replicate and the mean of all replicates was then calculated for each mouse.

**Quantitative real-time PCR.** Total RNA was isolated using RNeasy Mini Kit (Qiagen) after homogenization in RLT buffer (Qiagen) with zirconium oxide beads (1 mm; Thistle Scientific) in FastPrep-24™ 5G benchtop homogenizer (MP Biomedicals). A High Capacity cDNA Reverse Transcription Kit (Applied Biosystems) was used and quantitative real-time PCR was performed with an Applied Biosystems™ QuantStudio™ 7 Flex PCR system using 100 ng cDNA template, Taqman® Fast Universal PCR Master Mix and gene-specific Taqman® Gene expression assays (Human: *ACTA2* Hs00426835_g1, *CA9* Hs00154208_m1, *COL1A1* Hs00164004_m1, *GSTA2* Hs00747232_mH, *IGF1* Hs01547656_m1, *NOX4* Hs01379108_m1, *NQO1* Hs01045993_g1, *TGFB1* Hs00998133_m1, *TNFA* Hs00174128_m1; Mouse: *Ccl5* Mm01302427_m1, *Col1a* Mm00801666_g1, *Cybb* Mm01287743_m1, *Cxcl1* Mm04207460_m1, *Cxcl10* Mm00445235_m1, *Cxcl16* Mm00469712_m1, *Duox1* Mm01328685_m1, *Duox2* Mm01326247_m1, *Gsta2* Mm03019257_g1, *Hmox1* Mm00516005_m1, *Ifng* Mm01168134_m1, *Il22* Mm01226722_g1, *Nox1* Mm00549170_m1, *Nox4* Mm00627696_m1, *Nqo1* Mm01253561_m1, *Tgfb1* Mm01178820_m1, *Tnfa* Mm00443258_m1) (Applied Biosystems). Relative mRNA expression was calculated by ΔΔCT method and *GAPDH* Hs02786624_g1 or *Hprt* Mm03024075_m1 were used for normalization.

***C. rodentium* virulence associated genes.** Luminal content and cecal tissues were obtained from infected mice at 7 dpi. qPCR for *ler* and *escN* was performed using a SYBR Green Master mix with 30 ng (cecal content) or 100 ng (cecal tissue) of cDNA template and normalized to the expression of *gfp* in *C. rodentium* [25]. Primers were: *ler*, 5′-AAT ATA CCT GAT GGT GCT CTT G-3'and 5′-TTC TTC CAT TCA ATA ATG CTT CTT-3’; *escN*, 5′-CAG CCA TTT ACG CTT GGG GT-3′ and 5′-CGA CCA CGC TCA CCG ATA AG-3’; *gfp*, 5′-TTT CAA GAG TGC CAT GCC CG-3′ and 5′-CGT CTT GTA GTT CCC GTC-3’.

**Sircol assay for collagen quantification**. Tissue samples were dried at 37 °C overnight, weighed and homogenized (FastPrep-24™ 5G benchtop homogenizer; 1 mm zirconium oxide beads shaken for 2 cycles at 6 m/s for 30s with 5min rest periods) in 1 ml of 0.1 μg/ml pepsin in 0.5 M acetic acid and left at 4 °C overnight for pepsin digestion. Collagen extraction was performed using a commercial kit (Sircol™ Soluble Collagen Assay and Sircol™ Insoluble Collagen Assay, BioColor) according to the manufacturer's instructions. Collagen was quantified using absorbance at 550 nm correlated to a bovine collagen standard curve and expressed as μg collagen/mg dry tissue.

**Statistics.** Individual data points/symbols represent one mouse, one human subject, or one sample, and error bars represent mean ± SEM or median ± IQR as indicated. Statistical analysis was performed using GraphPad Prism 8 software. For data that were interval or ratio, normally distributed, and homoscedastic, differences between two or multiple groups were assessed using two-tailed unpaired *t*-test or one-way ANOVA (with Tukey correction for multiple comparisons) respectively. For non-homoscedastic data, two independent groups were compared using Welch's two-tailed *t*-test, and multiple independent groups were compared using Brown-Forsyth and Welch ANOVA tests (with Dunnet correction for multiple comparisons). Groups that were not independent were analyzed using two-way ANOVA (with Tukey correction for multiple comparisons within families). Data were assessed for normality and lognormality using D'Agostino & Pearson test. Where appropriate, non-normal data were normalized by transformation through Y = log(Y), re-assessed using D'Agostino & Pearson's normality test, then analyzed as described above. For interval/ratio data that were not normal and could not be normalized, and for ordinal or categorical data, groups were compared using the non-parametric Mann Whitney test for two independent groups, or Kruskall-Wallis with Dunn's multiple comparisons for multiple independent groups. Significance levels were graphically depicted as follows * p < 0.05, **p < 0.01, ***p < 0.001, ****p < 0.0001, ns: not significant.

**Study approval.** This study was approved by the Research Ethics Committees of St Vincent's University Hospital and of Children's Health Ireland. Written, informed consent from patients or guardians (for pediatric patients) was obtained prior to data and sample collection and analysis. Animal experiments were performed in accordance with EU Directive 2010/63/EU and were approved by the UCD Animal Research Ethics Committee and the Health Products Regulatory Authority of Ireland.

## 3 Results

### 3.1 *NOX4* expression is elevated in IBD specimen

**Fig. 1:**
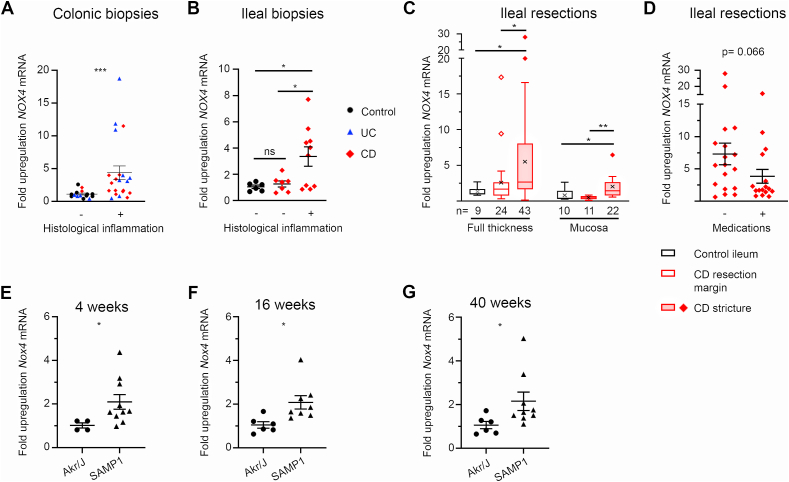
***NOX4* is upregulated in ileal strictures and inflamed mucosa.***NOX4* expression in mucosal (**A**) colonic biopsies and (**B**) ileal biopsies relative to non-IBD control biopsies. (**C**) Expression of *NOX4* in ileal strictures and surgical resection margins relative to non-IBD ileum and (**D**) *NOX4* expression in full thickness stricture specimens in patients on anti-inflammatory and/or immunosuppressive therapies compared to patients not on medication. Ileal *Nox4* expression in SAMP1/YitFc mice relative to Akr/J mice aged 4 weeks (**E**), 16 weeks (**F**) and 40 weeks (**G**). Data represented as (**A**, **B**, **D-G)** scatter plots showing mean ± SEM and (**C**) box-plot with Tukey whiskers and ‘x’ at mean. (**A**) by Mann Whitney test, (**B**) by Brown-Forsythe and Welch ANOVA with Dunnett's multiple comparisons test. (**C**, **D**) data were normalized by transformation (Y = log(Y)) then analyzed by two-factor ANOVA, with Tukey's multiple comparison test applied within full thickness and mucosal groups, (**E-G**) by unpaired two-tailed *t*-test. (**A**) n = 15 uninflamed (9 controls, 4 UC, 2 CD) and 20 inflamed biopsies (8 UC, 12 CD), (**B**) n = 6 controls, 7 uninflamed CD B1, 9 inflamed CD B1 biopsies.

Elevated NOX4 expression and H_2_O_2_ generation have been linked to acute and chronic inflammation in the vasculature, heart, lung, and liver, but the impact of NOX4 on intestinal disease and the development of fibrotic strictures is not known. We assessed *NOX4* expression in endoscopic biopsy specimens obtained from treatment-naïve pediatric patients, classified as CD, UC or non-IBD controls. Patients were phenotyped using the Paris classification of pediatric IBD according to disease diagnosis and behavior, and colonic and terminal ileal mucosal samples were categorized and further grouped into inflamed versus non-inflamed by clinical histopathological examination. *NOX4* was highly upregulated in intestinal tissues of CD and UC patients when inflammation was present (Fig. 1A and B). Strictures are uncommon in children, and thus the number of patients with stricturing CD was not sufficient to correlate *NOX4* expression with CD behavior profiles (B1 vs B2/B2B3 phenotype). To determine *NOX4* expression in intestinal strictures, specimens were obtained from children and adults undergoing ileal or ileo-colic resection, and non-IBD specimens were collected from adult colorectal cancer patients undergoing ileostomy formation or reversal, thus representing healthy ileal tissue. Demographic and clinical patient characteristics are listed in Supplemental Table 1. Full thickness and paired mucosal samples were taken from control ileum, CD resection margins and CD strictures and analyzed for *NOX4*, various profibrotic genes and NRF2 target gene expression. *NOX4* mRNA expression in full thickness and mucosal samples was upregulated in strictures compared to non-IBD ileal tissue (mean 5.5-fold), with significant variability both within strictures and between patients (Fig. 1C). Grouping patients according to their medication status (no medication vs anti-inflammatory/immunosuppressive medication) showed a trend to lower *NOX4* levels in patients receiving therapeutic interventions (Fig. 1D). Expression of profibrotic mediators *TGFB1*, *IGF1*, *COL1A1*, and *ACTA2* closely mirrored the upregulation of *NOX4,* with maximal levels in strictures (Supplemental Fig. 1A–H), while the pattern of *GSTA2* or *NQO1* expression, both NRF2 target genes, did not correlate with *NOX4* mRNA levels (Supplemental Fig. 2A–D). These results indicate either both a proinflammatory and profibrotic, or a host protective counter-regulatory role of NOX4 generated H_2_O_2_, thus necessitating comprehensive evaluation in animal models. SAMP1/YitFc mice are considered a highly relevant model of spontaneous CD-like ileitis which progresses to intestinal fibrosis and occasionally to stenosis by 40 weeks of age [28]. As environmental conditions can alter disease development SAMP1/YitFc mice were analyzed for ileal immune cell infiltration, villous atrophy, crypt abscesses, goblet cell hyperplasia and muscularis propria hypertrophy at different ages. First signs of ileitis were present in some mice at 4 weeks which is earlier than previously described, and all mice showed a progressive increase in prevalence, severity and anatomical extent of ileitis between 4 and 40 weeks of age. At 40 weeks of age terminal ileal sections of SAMP1/YitFc mice displayed extensive muscularis propria hypertrophy in combination with submucosa expansion and collagen deposition (Supplemental Fig. 3). This disease course permits the analysis of Nox4 expression at different stages of intestinal inflammation and fibrogenesis. A two-fold increase of *Nox4* expression was observed in SAMP1/YitFc mice compared to control Akr/J mice, independent of the disease stage (Fig. 1E–G). Thus, Nox4 levels remained elevated in intestinal inflammation without any significant increase during development and progression of fibrosis in this CD-like animal model. In wildtype C57BL/6J mice we reported elevated *Nox4* expression in acute chemical colitis [29] and observed a spike of *Nox4* levels at the onset of infectious colitis (not shown). We conclude that expression of *NOX4* is consistently elevated in human and murine colitis, during fibrogenesis and in strictures.

### 3.2 Nox4 deficiency aggravates *C. rodentium* pathogenesis

**Fig. 2:**
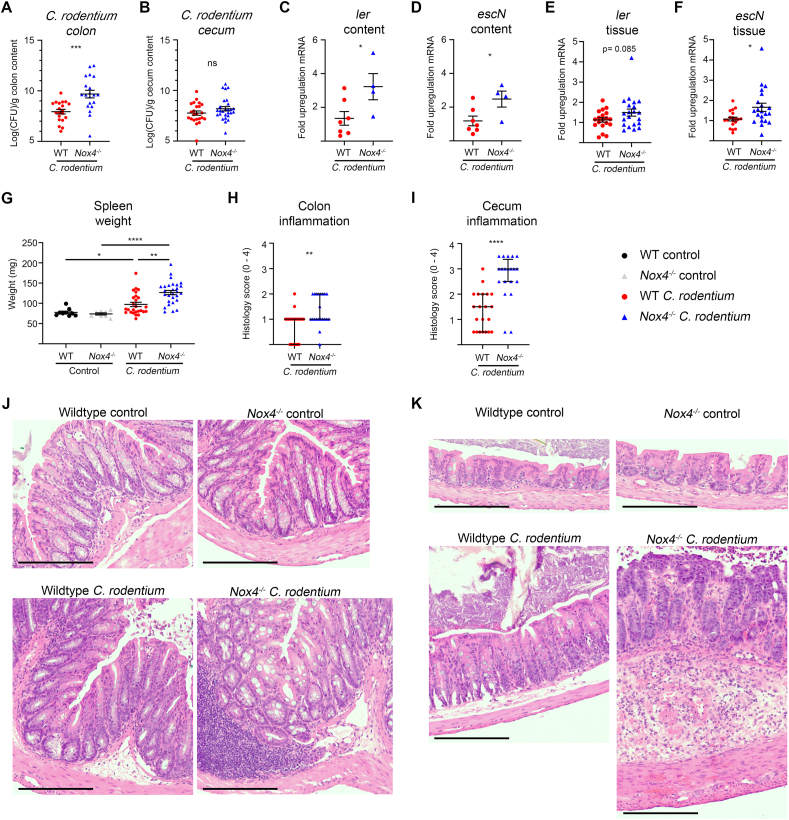
***C. rodentium* colitis is exacerbated in *Nox4***^***−/−***^**mice.** Mice were inoculated with *C. rodentium* (1 × 10^9^ CFU) and analyzed on d7. Bacterial load in colonic (**A**) and cecal content (**B**). Real time PCR of *C. rodentium* virulence gene expression in *Nox4*^*−/−*^ mice, normalized to wildtype infected mice, in cecal content (**C, D**) and cecal tissue (**E**, **F**), and (**G**) spleen weight. Histology inflammation scores for colon (**H**) and cecum (**I**). Representative colon (**J**) and cecal (**K**) sections stained with H&E; scale bar 200 μm. (**A-G**) represented as mean ± SEM. (**H**, **I**) represented as median ± IQR. (**A**, **B**) analyzed by unpaired two-tailed *t*-test on log-transformed data, (**C, D**) analyzed by unpaired two-tailed *t*-test, (**E**, **F**) analyzed by Welch's two tailed *t*-test, (**G**) analyzed by Brown-Forsythe and Welch ANOVA with Dunnett's test for multiple comparisons, (**H**, **I)** analyzed by Mann Whitney test between wildtype and *Nox4*^*−/−*^ treated mice. Data represent four independent experiments.

NOX4 generated H_2_O_2_ is a long-range signaling mediator, which has been linked to harmful oxidative modifications under certain conditions, but may provide protective counterbalance functions when homeostasis is disturbed. To discern the function of Nox4 in intestinal inflammation in more detail, the response of *Nox4*^*−/−*^ mice and matched wildtype mice to oral infection with *Citrobacter rodentium*, a murine pathogen mimicking human EPEC infection and leading to crypt hyperplasia and resolving bacterial colitis [30], was studied. *C. rodentium* colonization at day 7 was substantially increased in the colon but not in the cecum of Nox4-deficient mice (Fig. 2A and B). Yet, virulence factors encoded in the *C. rodentium* LEE pathogenicity island such as *ler* and *escN* were upregulated in luminal content and to a lesser extent in cecal tissue of these mice (Fig. 2C–F), and spleen weight was increased, suggesting microbial dissemination (Fig. 2G). Histological examination indicated minimal cellular infiltration in the colonic mucosa and cecal crypt hyperplasia in wildtype mice. Cell infiltration in colonic tissue was significantly increased in *Nox4*^*−/−*^ mice, forming large foci, while cecal crypt hyperplasia was accompanied by massive mucosal and submucosal inflammatory cell infiltration (Fig. 2H–K). Early events such as neutrophil chemotaxis induced by generation of *Cxcl1*, and upregulation of *Il22*, *Reg3g* and *Ifng* were maintained and even enhanced in Nox4-deficient mice (Supplemental Fig. 4A–D), thus interference with these host defense mechanisms cannot explain the exaggerated tissue pathology.

### 3.3 *Nox4*^*−/−*^ mice develop severe chemically-induced colitis

**Fig. 3:**
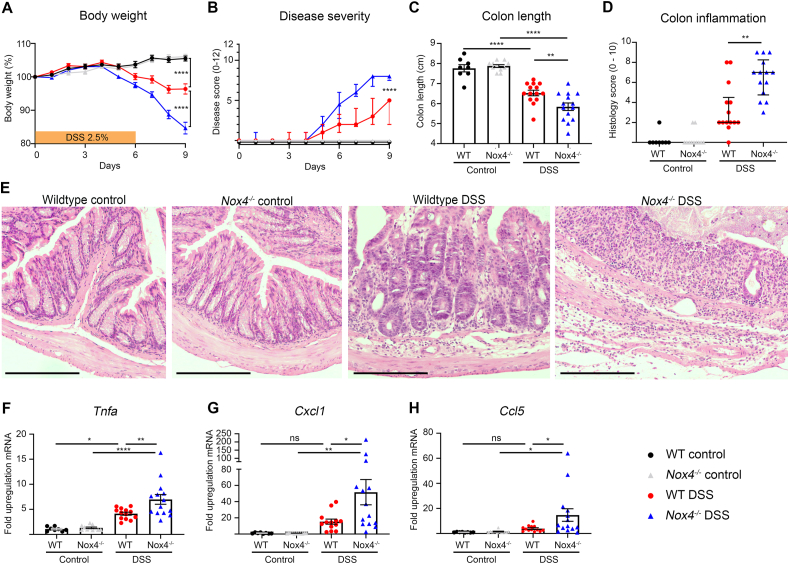
***Nox4***^***−/−***^**mice develop more severe DSS colitis than wildtype mice.** Mice were treated for 6d with 2.5% DSS and analyzed on d9. (**A**) Body weight curve, (**B**) disease severity score, (**C**) colon length, (**D**) histological inflammation, (**E**) representative colon sections stained with H&E; scale bar 200 μm. Real time PCR data depicting fold upregulation of mRNA in *Nox4*^*−/−*^ mice compared to wildtype mice for (**F**) *Tnfa*, (**G**) *Cxcl1* and (**H**) *Ccl5*. (**A**, **C**, **F**–**H**) represented as mean ± SEM, (**B**, **D**) represented as median ± IQR. (**A**) analyzed by one-way ANOVA on day 9, (**B**, **D**) analyzed by Mann Whitney test between wildtype and *Nox4*^*−/−*^ DSS groups on day 9, (**C**) analyzed by one-way ANOVA with Tukey's multiple comparisons test, (**F**–**H**) data were log-transformed to a normal distribution then analyzed by ordinary one-way ANOVA with Tukey's multiple comparisons test. Data represent two independent experiments.

Using combinations of various Nox isoform inactivation and knockout mouse strains we observed that maintaining catalytically active Nox4 delayed the mortality rate of dextran sodium sulfate (DSS) treated, Nox1-3 inactivated mice [29]. To study this protective phenotype in more detail wildtype mice and *Nox4*^*−/−*^ mice were exposed to DSS for 6 days, which resulted in accelerated body weight loss and increased disease severity in Nox4-deficient mice (Fig. 3A and B). Characteristic indicators of severe inflammation including colon shortening, histological inflammation and *Tnfα* levels were significantly augmented in *Nox4*^*−/−*^ mice (Fig. 3C–F). While wildtype mice exhibited crypt hyperplasia, scattered mucosal inflammatory cell infiltration and minimal crypt injury at the disease apex, complete destruction of entire crypts and sizeable foci of inflammatory cells leading to extensive transmural inflammation were present in *Nox4*^*−/−*^ mice (Fig. 3E). Loss of Nox4 increased the levels of neutrophil attracting chemokines *Cxcl1* and *Ccl5* (Fig. 3G and H), suggesting augmented neutrophil recruitment as initial trigger for subsequent tissue injury. Thus, in two acute colitis models the absence of Nox4 did not reduce inflammation but amplified tissue damage.

### 3.4 No attenuation of pathogen-induced fibrosis in *Nox4*^*−/−*^ mice

**Fig. 4:**
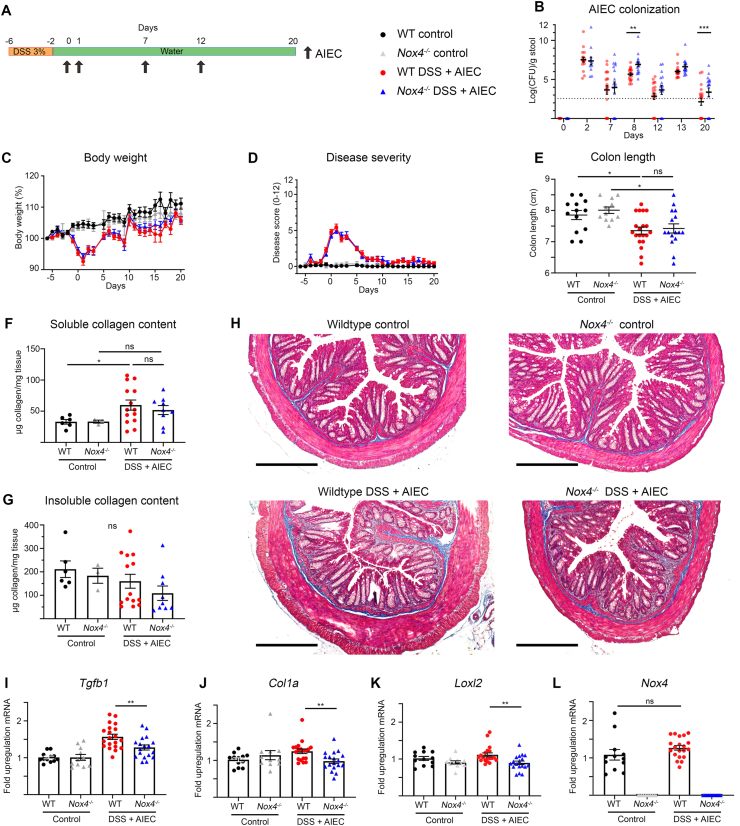
**AIEC-induced intestinal fibrosis is not attenuated in *Nox4***^***−/−***^**mice.** Mice were exposed for 4d to 3% DSS (or water) before repeated oral administration of AIEC (1 × 10^9^ CFU, arrow) (or PBS as control) (**A**). (**B**) Quantification of AIEC in feces of *Nox4*^*−/−*^ mice compared to wildtype mice; dotted line represents detection limit. (**C**) Body weight loss and (**D**) disease severity scoring. Colon inflammation and fibrosis at d20 was assessed by (**E**) colon length, (**F**, **G**) soluble and insoluble collagen, and (**H**) Masson trichrome stained sections with collagen in blue; scale bar 300 μm, and qPCR on colonic mRNA, normalized to wildtype mice, of (**I**) *Tgfb1*, (**J**) *Col1a*, (**K**) *Loxl2*, and (**L**) *Nox4*. (**B**, **C**, **E-G**, **I-L**) represented as mean ± SEM, (**D**) represented as median ± IQR, (**B**) data were log-transformed then analyzed with multiple unpaired t tests with Holm-Sidak correction, (**E-G**) data were analyzed by one-way ANOVA with Tukey's multiple comparisons test, (**I**, **J**, **K**) by unpaired *t*-test between wildtype and *Nox4*^*−/−*^ treated groups, or (**L**) Welch's *t*-test between wildtype groups. Data represent three independent experiments.

Acute inflammation initiates fibrogenesis, but distinct signaling pathways and mechanisms may perpetuate fibrogenesis even in the absence of inflammation [31]. Evaluation of these mechanisms in mice requires repeated exposure models. Bacterial infections are considered a risk factor, initial trigger or propagating mechanism for chronic intestinal inflammation [32]. The high prevalence of adherent-invasive *E. coli* (AIEC) in the intestinal mucosa of CD patients and the fibrotic immunopathology reported in mice after oral AIEC infection guided our choice of using continuous pathogen colonization as intestinal fibrosis model [33,34]. The AIEC strain NRG587c displayed high host fitness in mice and led to a profibrotic phenotype in the colon and cecum of C57BL/6 mice, but persistent colonization necessitated antibiotic pretreatment [34,35]. In order to avoid significant drug-induced changes of the indigenous microbiota a short DSS pretreatment, mimicking IBD-like inflammatory processes, was followed by several rounds of oral AIEC administration (Fig. 4A). In this modified fibrosis model stable AIEC colonization was achieved at all time points, resulting in higher AIEC burden in *Nox4*^*−/−*^ mice (Fig. 4B). *Nox4*^*−/−*^ mice and wildtype mice showed similar body weight loss and colitis severity during the 20-day disease course (Fig. 4C and D). At the end point inflammation was only present in the colon, independent of the genotype (Fig. 4E, data not shown). This modified DSS/AIEC model resulted in colonic and cecal fibrosis as shown by Masson's trichrome staining of collagen deposition in tissue sections and by soluble collagen quantification, but no significant differences were apparent between wildtype and Nox4 knockout mice (Fig. 4F, H, and Supplemental Fig. 5). The DSS/AIEC fibrosis model did not lead to a significant rise in insoluble collagen in either mouse strain (Fig. 4G and Supplemental Fig. 5), and fibrosis-associated *Tgfb, Col1a, Loxl2* and *Nox4* genes were either only slightly increased or not altered, and only minimally downregulated in *Nox4*^*−/−*^ mice (Fig. 4I-L and Supplemental Fig. 5). Experimental end point analysis of mouse ceca revealed an even more profound increase in soluble collagen but no particular upregulation of other fibrotic markers, and no substantial differences between wildtype and *Nox4*^*−/−*^ mice. Fibrosis was not detected in ilea of either mouse strain (Supplemental Fig. 5). Fibrogenesis was less prevalent in the DSS/AIEC model compared to AIEC administration after antibiotic pretreatment [35], which may reflect indigenous microbiota-induced changes, effects of the antibiotic, or environmental differences in facilities. In conclusion, Nox4-generated H_2_O_2_ is beneficial for the host in decreasing pathogen colonization but has only minimal impact on collagen deposition in this fibrosis model.

### 3.5 Nox4 promotes recovery but not fibrogenesis in chronic colitis

**Fig. 5:**
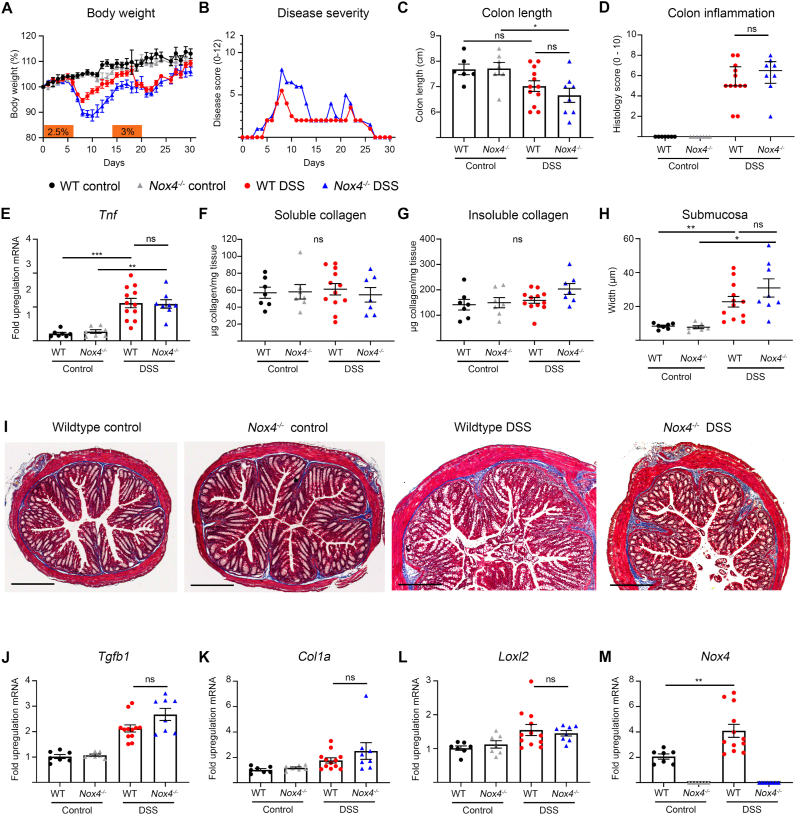
**Colonic fibrosis after repeated DSS exposure is not altered in *Nox4***^***−/−***^**mice.** Mice were treated with two cycles of 6d DSS (2.5%, 3%) and recovery (8d, 10d), and analyzed on d30. (**A**) Body weight loss, (**B**) disease severity scores, (**C**) colon length, (**D**) colon histological inflammation, (**E**) real time PCR of *Tnf* expression, (**F, G**) soluble and insoluble collagen quantification, (**H**) submucosa width determination, (**I**) representative colon sections stained with Masson trichrome with collagen in blue; scale bar 300 μm qPCR depicting fold upregulation of mRNA in *Nox4*^*−/−*^ mice normalized to wildtype mice of (**J**) *Tgfb1*, (**K**) *Col1a*, (**L**) *Loxl2* and (**M**) *Nox4*. (**A**, **C**, **E-H**, **J-M**) represented as mean ± SEM, (**B**) represented as median and (**D**) represented as median ± IQR. (**C**, **E**, **F**, **G, H**, **L**) Data were analyzed with ordinary one-way ANOVA with Tukey's correction for multiple comparisons, (**D**) Mann Whitney test between treated wildtype and *Nox4*^*−/−*^ groups, (**J, K**) analyzed by Brown-Forsythe and Welch ANOVA with Dunnett's multiple comparisons test, (**M**) analyzed by Welch's *t*-test between wildtype groups. Data represent two independent experiments.

Repeated inflammatory insults induce aberrant wound healing and serve as profibrotic stimulus. Increased collagen deposition and muscularis propria thickening was observed in mice after several on-off cycles of DSS or TNBS [36]. In acute DSS colitis loss of Nox4 caused extensive tissue damage (Fig. 3), but it was not evident from these results whether a) mice could recover from the insult, b) enhanced inflammation would drive fibrogenesis or c) Nox4 is required for distinct fibrogenic pathways independent of proinflammatory processes. Exposure to two cycles of DSS separated by recovery phases showed, as expected, increased disease severity in *Nox4*^*−/−*^ mice during the first colitis cycle (weight loss, disease index) followed by a slower recovery, while the disease course and recovery of wildtype and *Nox4*^*−/−*^ mice after the second DSS exposure was comparable (Fig. 5A and B). At the endpoint, hallmarks of inflammation including shortened colon length, histological colon inflammation and increased *Tnfa* expression were observed in wildtype mice and *Nox4*^*−/−*^ mice without any discernible difference between strains (Fig. 5C–E). The main connective tissue phenotype was the increased thickness of the submucosa and upregulation of *Tgfb1* expression, while neither soluble nor insoluble collagen, nor *Col1a* and *Loxl2* were substantially elevated (Fig. 5F-L). Even though *Nox4* expression was upregulated in a comparable manner as observed in SAMP1/YitFc mice (Fig. 5M), the deletion of Nox4 did not alter any fibrosis parameters.

## 4 Discussion

Enhanced ROS levels are typically associated with inflammation and tissue injury. In the gastrointestinal tract excessive intestinal epithelial cell (IEC) death and loss of barrier function are hallmarks of acute inflammation. During this phase oxidative modifications on proteins and lipids are commonly noted, but the enzymatic source(s) of these oxidants have not yet been fully determined. Phagocyte NADPH oxidase NOX2-derived oxidants such as hypochlorous acid or peroxynitrite and mitochondria-derived ROS are often implicated in triggering inflammatory processes. The interplay between persistently elevated ROS and intestinal pathophysiology is complex and not well understood. This stems partly from the diverse reactivity of generated oxygen/nitrogen species, their location-dependent effects, cooperation of several ROS sources, and concurrent signal transmission with feed-forward and feed-back loops. For example, TNFα, a critical driver of IEC shedding in colitis, induces superoxide generation via NOX2, NOX1 and the mitochondria, which will trigger, depending on the context, apoptosis, necroptosis, survival and further upregulation of proinflammatory cytokines such as TNFα and IL-6 [37]. ROS generation by NOX or mitochondria is also involved in Toll-like receptor signaling and in inflammasome activation, leading to the production of IL-18, IL-1β and other cytokines [38]. However, loss-of-function variants in NOX2 complex components that decrease or abrogate superoxide generation induce hyperinflammation in chronic granulomatous disease (CGD) patients with 40–50% of patients developing CGD-IBD over time [39,40]. Similarly, decreased superoxide or H_2_O_2_ generation by NOX1 and DUOX2 variants has been associated with chronic inflammation in very early onset IBD patients [41]. These and many other observations point to a homeostatic range of ROS as essential for maintaining intestinal health, while persistently increased or decreased ROS levels perpetuate inflammation.

NOX4, an oxidase primarily regulated by transcription, constitutively produces the less reactive, relatively stable second messenger H_2_O_2_. While H_2_O_2_ can be converted to highly reactive secondary metabolites, its main purpose is redox signaling. NOX4's catalytic activity propagates signals involved in disease progression, for example in tumorigenesis, but also promotes pathways supporting homeostasis and restitution. Hence, the effect of NOX4 upregulation may be detrimental or beneficial in various cell types and under different conditions. Here we show that *NOX4* expression was significantly increased in inflamed tissues of IBD patients and in acute intestinal inflammation in mice. Removing Nox4 in murine acute chemical and bacterial colitis disabled protective host mechanisms, resulting in severe tissue destruction and protracted recovery. Barrier protection, host defense and tissue restitution in the intestine have been commonly linked to the epithelial NADPH oxidases Nox1 and Duox2 with further reinforcement by Nox2 expressed in innate immune cells resident or recruited to the lamina propria [42]. Nox4 deficiency did not result in substantial changes in the expression levels of cecal or colonic epithelium-associated *Nox1*, *Duox2*, or *Duox1*, or neutrophil/macrophage-associated *Cybb* (Nox2) in homeostasis, or in pathogen or chemically induced acute colitis, and the general pattern of Nox/Duox upregulation at the peak of inflammation was maintained (Supplemental Figs. 6 and 7). These results suggest a more generalized failure of yet unidentified defensive counter-regulatory mechanisms in the absence of Nox4.

Multifaceted protection of intestinal barrier function is often provided by the Keap1-NRF2 pathway which induces antioxidant response element (ARE)-containing genes involved in redox balance, metabolism and inflammation [[43], [44], [45]]. In certain conditions NOX4 is coupled to the Keap1-NRF2 pathway, acting as a redox rheostat by inducing NRF2-dependent gene transcription [46]. This scenario was described in a model of acute kidney injury where Nox4 deficiency resulted in augmented tubular cell apoptosis due to a decline in the antioxidant and antiapoptotic response [47]. Similarly, the protective role of NOX4 in the vasculature was linked to activation of the NRF2 pathway [48,49]. Analysis of NRF2 target genes involved in ROS detoxification (*Nqo1*, *Gsta2*) and heme/iron metabolism (*Hmox1*) at the apex of acute murine colitis shows a more nuanced picture with all three genes being differentially affected by Nox4 deficiency (Supplemental Fig. 8). Only the expression of NAD(P)H quinone dehydrogenase 1 (Nqo1) was significantly downregulated in the colon of DSS-treated *Nox4*^*−/−*^ mice. Similarly to *Nox4*^*−/−*^ mice, Nqo1-deficient mice showed aggravated DSS-induced mucosal damage [50] and the emerging role of Nqo1 as redox sensitive protein and mRNA binding partner suggests that the Nox4-dependent regulation of Nqo1 during inflammatory challenge may have multiple downstream consequences [51]. Mechanistic insights into the redox sensitive activation of NRF2 and its target genes are still limited and likely context-dependent as in certain pathologies NOX4 activity was decoupled from the NRF2-initiated antioxidant response [46]. In addition, the protective role of NOX4 in our study extended to improved host defense against the intestinal pathogens *C. rodentium* and AIEC by reducing bacterial colonization, either due to exposure to NOX4-derived H_2_O_2_ [52,53] or via NOX4-dependent redox signaling affecting other antibacterial defense systems.

Clinical observations support the view that inflammation is a prerequisite for the initiation of intestinal fibrosis, but neither inflammation alone nor the extent of inflammation seem to correlate well with the progression of fibrosis [54,55]. Once initiated, intestinal fibrogenesis may become an independent, self-perpetuating process, which would explain why the advent of highly effective anti-inflammatory biological agents seems not to have significantly reduced the incidence of fibrotic CD [56]. Thus, while the presence of NOX4 is beneficial in acute colitis, elevated H_2_O_2_ levels due to NOX4 upregulation may drive intestinal fibrosis, similar to observations in lung and liver fibrosis [10,11,13,14,16]. To address this question, we compared mice with global deficiency in Nox4 to wildtype mice, using F0, F2 and F3 generation mice to account for putative microbiota differences that may influence fibrosis development. In both a chronic model of bacterial infection and a model with repeated cycles of epithelial injury, markers of fibrosis and remodeling did not differ substantially between genotypes or *Nox4*^*−/−*^ generations. Fibrosis in animal models is commonly determined by (semi)quantitative measurement of collagen deposition, profibrotic gene expression and myofibroblast infiltration. However, the dominant feature of intestinal fibrosis and stenosis in the CD-like SAMP1/YitFc ileitis model as well as in induced murine fibrosis models (active TGFB1 adenoviral transduction, *Ship*^*−/−*^, *Il10*^*−/−*^) is hypertrophy of the muscularis propria with only minimally increased extracellular matrix. In contrast, human ileal strictures combined muscular hypertrophy with dense collagen deposition in a significantly expanded submucosa (Supplemental Fig. 9), similar to observations by others [57,58]. Thus, existing mouse models of intestinal fibrosis can only recapitulate certain aspects of human intestinal fibrosis.

While our studies indicate that global deletion of *Nox4* did not alter intestinal fibrosis in mice, inducing kidney fibrosis by unilateral ureteral obstruction (UUO) or cardiac interstitial fibrosis by chronic pressure overload revealed protective effects of Nox4 in murine fibrogenesis [[59], [60], [61], [62]]. Overexpression of Nox4 in tubular cells did not induce kidney injury or modify UUO-induced lesions, while cardiomyocyte-specific Nox4 overexpression reduced interstitial fibrosis [59,63]. At present these diametrically different organ- and context-dependent pro- or antifibrotic effects of Nox4 are not understood, but it was recently suggested that the different expression levels of endogenous Nox4 in various cell types may influence the outcome. In the gastrointestinal tract several fibroblast subsets, smooth muscle cells, and cell undergoing transition to a mesenchymal phenotype such as intestinal epithelial cells and endothelial cells contribute to fibrogenesis [31]. All of these cell types likely express Nox4 at different quantities in homeostasis and during disease progression. The scarcity of sensitive, Nox4-specific antibodies hinders progressive analysis of intestinal tissue during murine fibrogenesis, and even with our validated anti-human NOX4 antibodies the classification of specific NOX4 expressing cell types in human stricture tissue was not convincingly achieved. Involvement of several mesenchymal cell populations was reported in inflamed UC patient biopsies and acute DSS colitis [64], but detailed studies with tissue specific Cre deleter mice will be necessary to identify Nox4 expressing cell types driving the extensive tissue injury in the acute inflammatory phase of colitis. Observations by us and others indicate that manipulating redox signaling can be a double-edged sword and that therapeutic approaches need to be tailored to particular conditions and patient populations to maximize clinical benefit while minimizing risk.

## Author contributions

ES, BB, SH and UGK developed the concept and designed the study. ES, GA, AS, MMD performed experiments and data analysis. SH, SM, DW and BS assisted ES with patient specimen collection. ES and UGK wrote the manuscript.

## Declaration of competing interest

The authors declare that they have no known competing financial interests or personal relationships that could have appeared to influence the work reported in this paper.
