# Supplementary material for: NADPH oxidase 4 is protective and not fibrogenic in intestinal inflammation
Source: Redox Biol. 2020 Oct 7;37:101752. doi: 10.1016/j.redox.2020.101752 (PMC7567035; doi:10.1016/j.redox.2020.101752)
Supplement: Multimedia component 1 [file mmc1.pdf]

Supplemental Information

NADPH oxidase 4 is protective and not fibrogenic in intestinal inflammation

Emily Stenke[1], Gabriella Aviello[1,5], Ashish Singh[1], Sean Martin[2], Des Winter[2], Brian Sweeney[3], Michael McDermott[3], Billy Bourke[1,3], Seamus Hussey[3,4], Ulla G Knaus[1,3*]

[1] Conway Institute, School of Medicine, University College Dublin, Dublin, Ireland

[2] St. Vincent's University Hospital, Dublin, Ireland

[3] National Children's Research Centre, Children's Health Ireland, Dublin, Ireland

[4] RCSI University of Medicine and Health Sciences, Dublin, Ireland

[5] present address: Department of Pharmacy, University of Naples Federico II, Naples, Italy

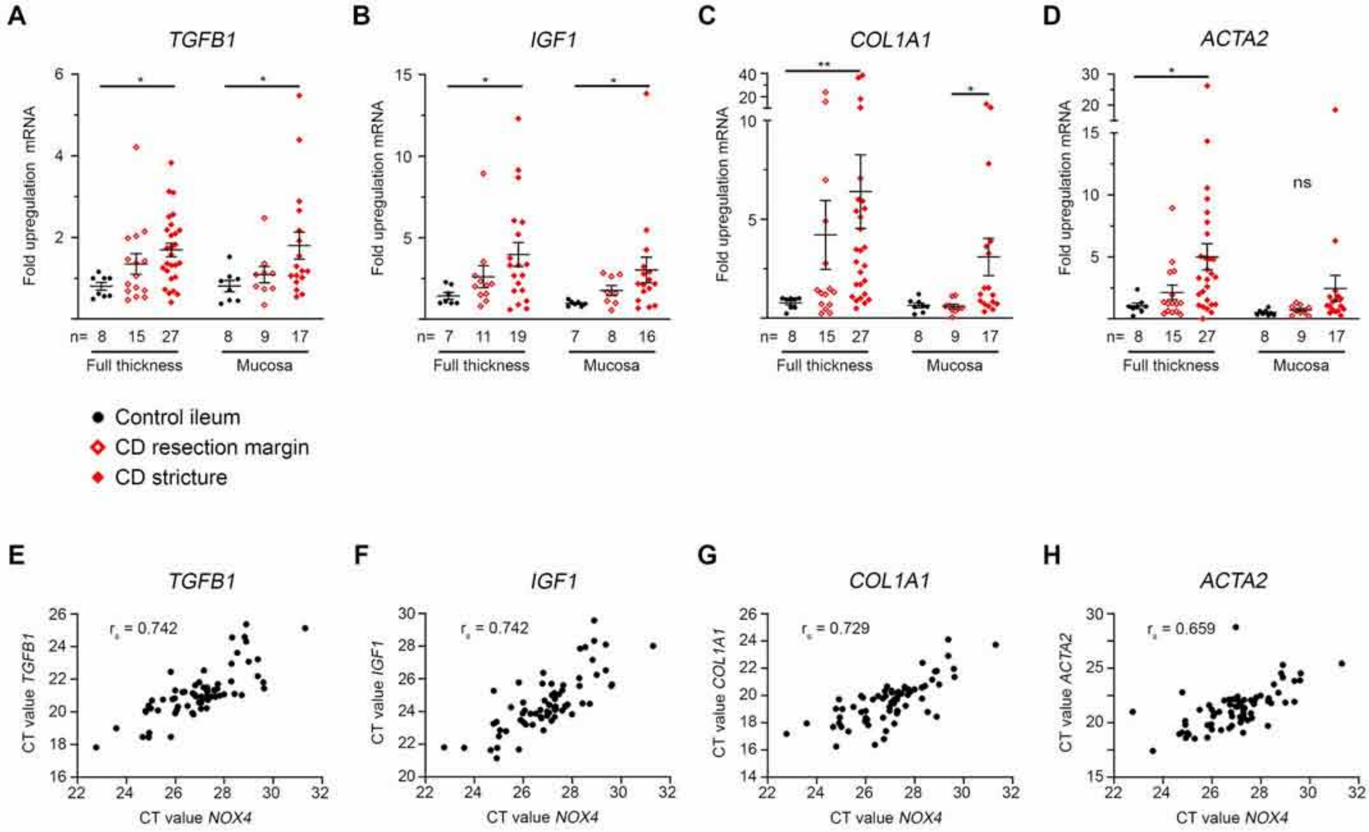

Supplemental Figure 1. *NOX4* expression in ileal strictures strongly correlates with markers of fibrosis. (**A-D**) *TGFB1*, *IGF1*, *COL1A1* and *ACTA2* mRNA expression in stricture tissue (filled red diamond) and resection margin (empty red diamond), compared to control ileum (black circle). (**E-H**) qPCR CT values for *NOX4* are plotted against CT values of genes of interest, for all samples combined. (**A-D**) Error bars represent mean ± SEM, data were normalized by transformation (Y=log(Y)) then analyzed by two-factor ANOVA with Tukey's multiple comparison test applied within full thickness and mucosal groups. (**E-H**) Data analyzed by Spearman's rank correlation coefficient (r_s). One data point represents one tissue sample.

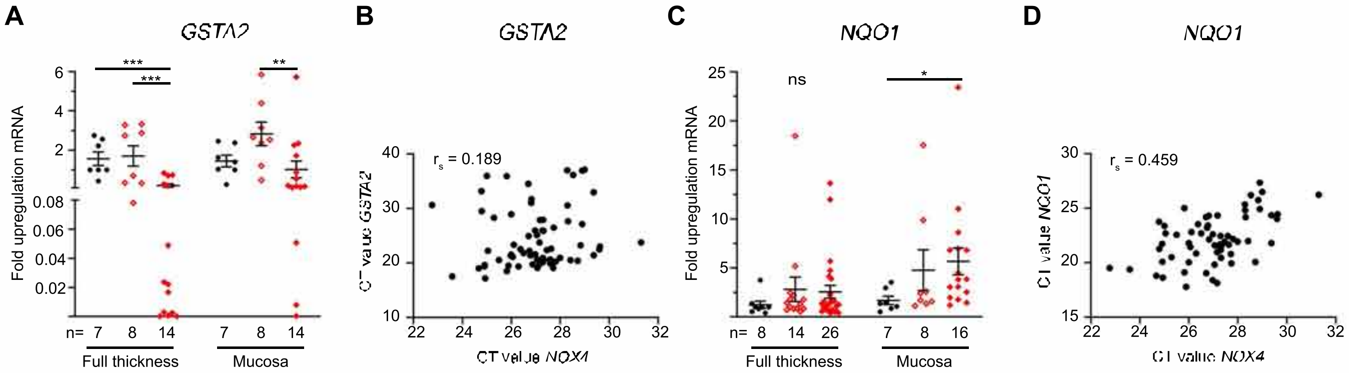

Supplemental Figure 2. ARE-targeted genes are differentially expressed in CD strictures. Expression of (**A**) *GSTA2* and (**C**) *NQO1* in stricture tissue (filled red diamond) and resection margin (empty red diamond), compared to control ileum (black circle). (**B, D**) qPCR CT values for *NOX4* are plotted against CT values of *GSTA2* and *NQO1* for all samples combined. (**A, C**) represented as mean ± SEM, data were normalized by transformation (Y=log(Y)) then analyzed by two-factor ANOVA with Tukey's multiple comparison test applied within full thickness and mucosal groups. (**B, D**) Data analyzed by Spearman's rank correlation coefficient (r_s). One data point represents one tissue sample.

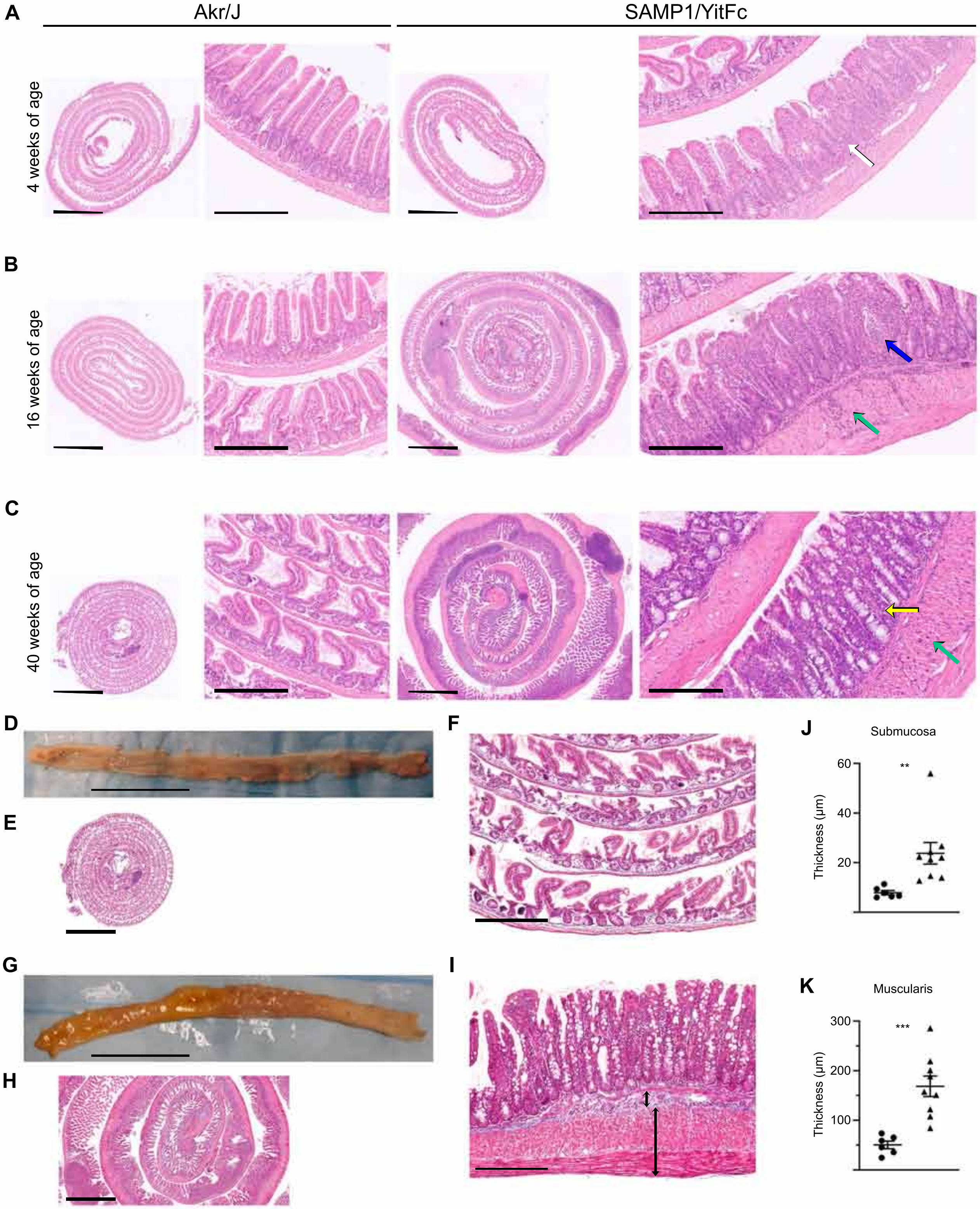

Supplemental Figure 3. Ileitis, an early feature of disease in SAMP1/YitFc mice, progresses to fibrosis by 40 weeks of age. Histological inflammation on H&E stained ileal sections of Akr/J and SAMP1/YitFc mice aged 4 weeks (**A**), 16 weeks (**B**), and 40 weeks (**C**). Crypt hyperplasia (white arrow), transmural inflammatory cell infiltration (green arrow), crypt abscess (blue arrow), and crypt hyperplasia with villous atrophy (yellow arrow) are shown. Fibrosis was assessed in 40 week old mice. Gross specimens of opened terminal ileum of Akr/J (**D**) and SAMP1/YitFc (**G**) were embedded in Swiss roll orientation and stained with Masson trichrome showing collagen in blue. Representative images at low (**E, H**) and high magnification (**F, I**). Quantitation of submucosa (**J**) and muscularis propria thickness (**K**) in SAMP1/YitFc mice (triangles) compared to Akr/J mice (circles). Scale bars represent 2 cm (**D, G**), 2 mm (low magnification images **A-C, E, H**) and 300 µm (high magnification images **A-C, F, I**). Images are representative of 4 Akr/J mice and 8 SAMP1/YitFc mice aged 4 weeks (**A**), 9 Akr/J and 8 SAMP1/YitFc mice aged 16 weeks (**B**), and 6 Akr/J and 9 SAMP1/YitFc mice aged 40 weeks (**C-I**). (**J, K**) Data represented as mean ± SEM, analyzed by Welch's unpaired t test.

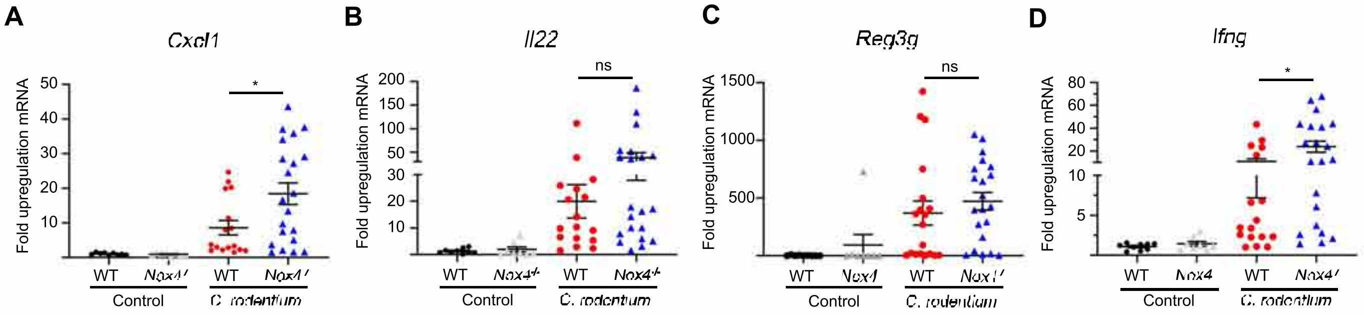

Supplemental Figure 4. Selected host responses in *C. rodentium* infection are maintained or increased in *Nox4*$^{-/-}$ mice. Quantitative PCR of cecal tissue for *Cxcl1* (**A**), *Il22* (**B**), *Reg3g* (**C**) and *Ifng* (**D**). (**A-D**) represented as mean ± SEM and analyzed by Mann Whitney test between wildtype (red circles) and *Nox4*$^{-/-}$ (blue triangles) treated mice. Data represent four independent experiments.

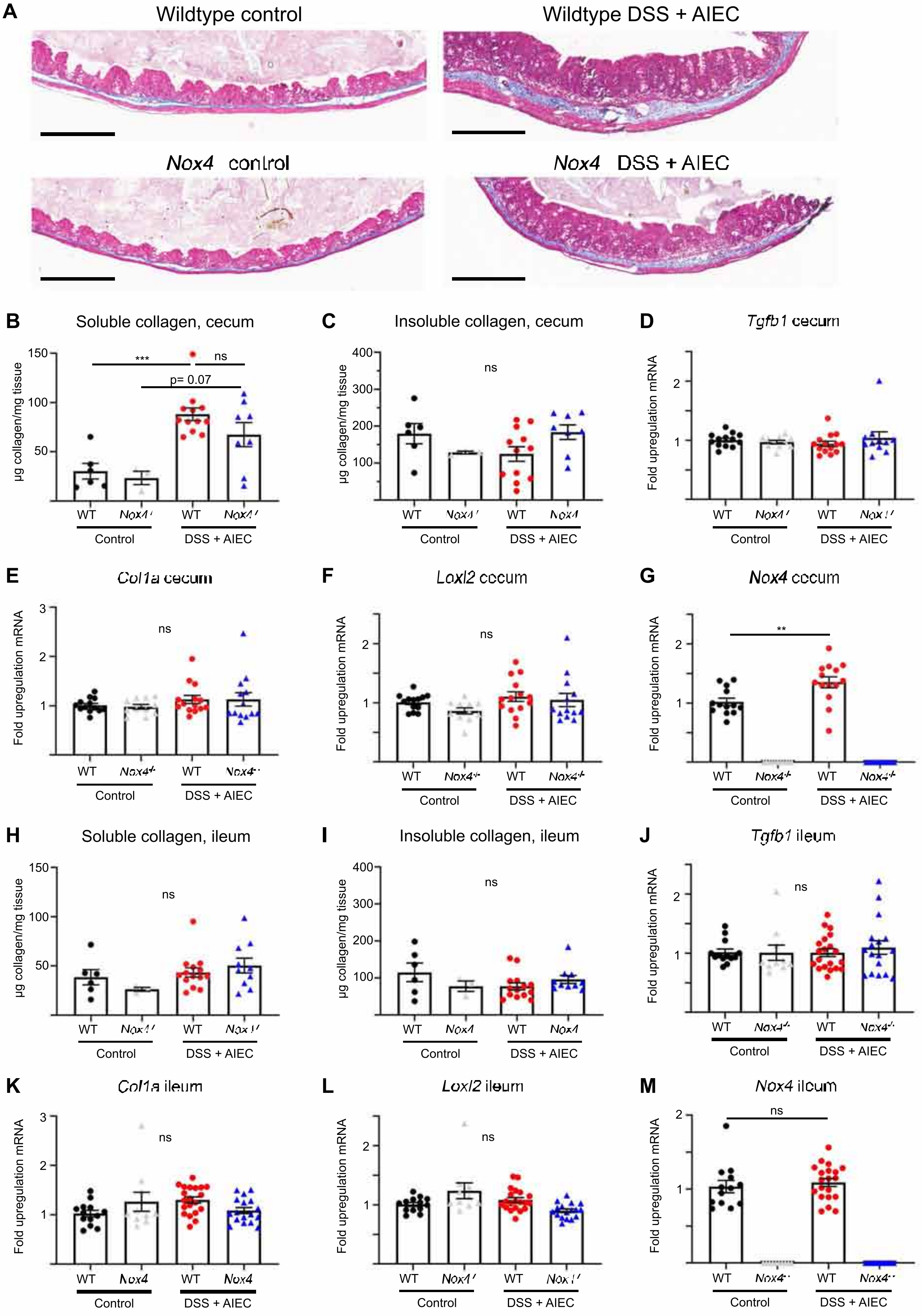

Supplemental Figure 5. Cecal fibrosis following DSS/AIEC treatment is Nox4 independent. Fibrosis at day 20 was assessed by Masson trichrome staining of cecum (**A**), soluble and insoluble collagen quantification in cecum (**B, C**) and ileum (**H, I**) and by qPCR of *Tgfb1*, *Col1a*, *Loxl2* and *Nox4* in cecum (**D-G**) and ileum (**J-M**). (**B-M**) error bars represent mean ± SEM and data analyzed by one-way ANOVA with Tukey's multiple comparisons test. Data represent three independent experiments.

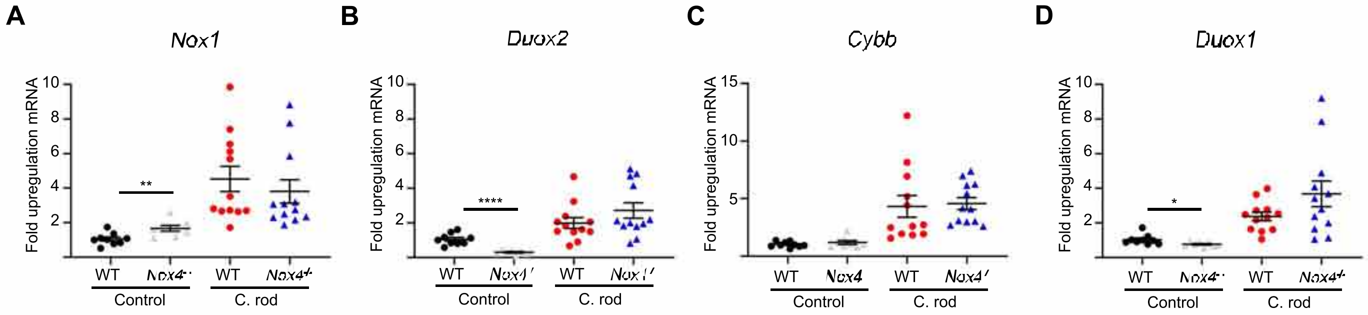

Supplemental Figure 6. Nox4 deficiency affects steady state cecal NADPH oxidase expression. Quantitative PCR of *Nox1*, *Duox2*, *Cybb* and *Duox1* (**A-D**) in cecal tissue on day 7 after inoculation with 1 x 10^9 CFU of *C. rodentium* (or PBS as control). (**A-D**) error bars represent mean ± SEM and data was analyzed by unpaired t tests between wildtype and *Nox4*$^{-/-}$ mice. Differences not statistically significant unless indicated otherwise. Data represent three independent experiments.

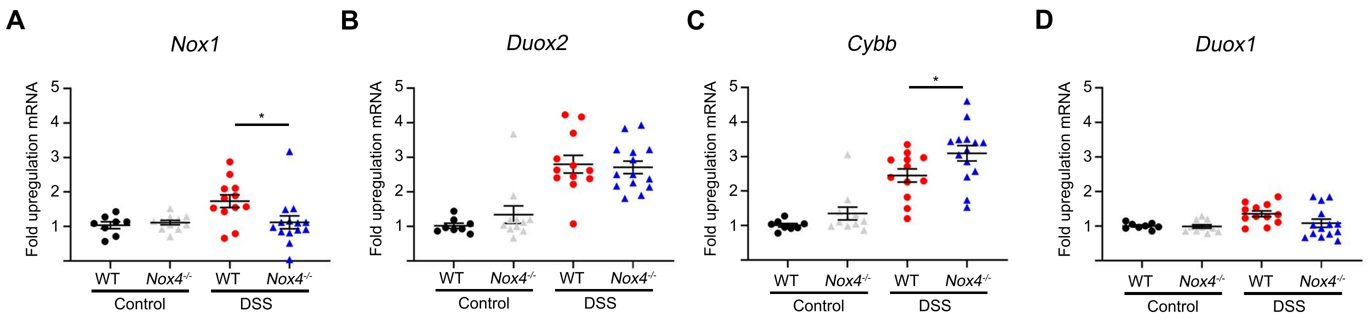

Supplemental Figure 7. Nox4 deficiency does not alter steady state and only marginally alters DSS colitis-induced colonic NADPH oxidase expression. Quantitative PCR of *Nox1*, *Duox2, Cybb* and *Duox1* (**A-D**) in colon tissue on day 9 of acute DSS colitis. (**A-D**) error bars represent mean ± SEM and data analyzed by unpaired t tests between wildtype and *Nox4*$^{-/-}$ mice. Differences not statistically significant unless indicated otherwise. Data represent two independent experiments.

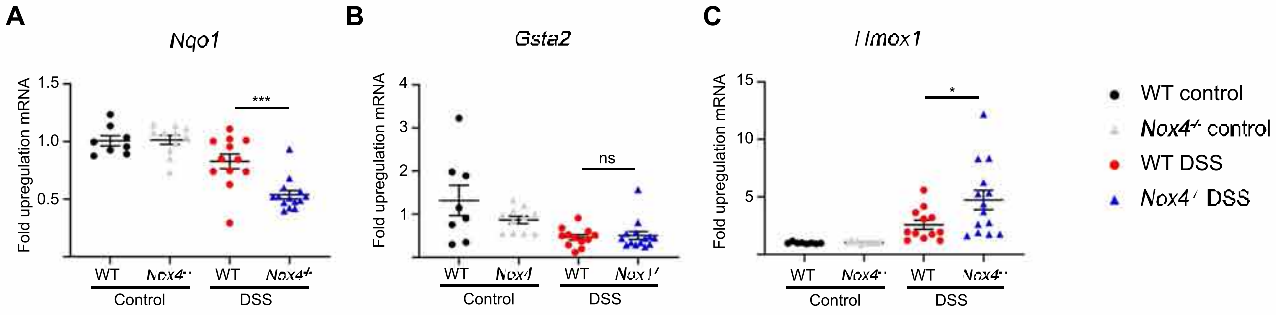

Supplemental Figure 8. Nox4 deficiency alters antioxidant responsive element (ARE)- targeted gene expression in DSS colitis. Quantitative PCR of *Nqo1*, *Gsta2* and *Hmox1* (**A-C**) in colon tissue on day 9 of acute DSS colitis. (**A-C**) error bars represent mean ± SEM and data analyzed by one-way ANOVA with Tukey's multiple comparisons test. Data represent two independent experiments.

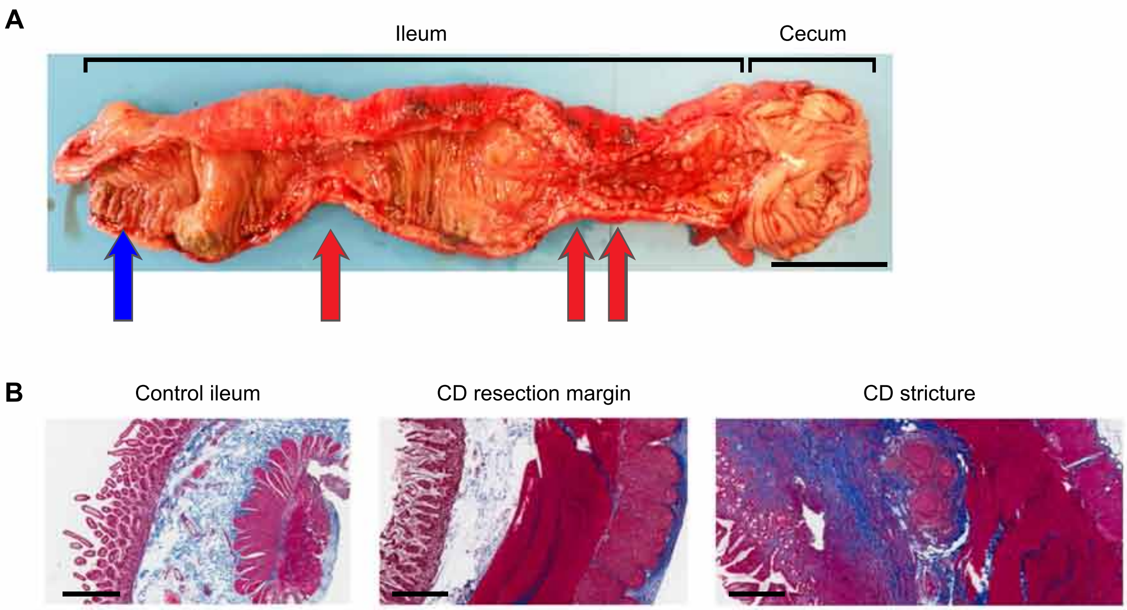

Supplementary Figure 9. Gross and microscopic appearance of an ileal stricture. (A) Photograph of a surgical resection specimen, containing two distinct strictures with co-existing inflammation. For each resection specimen, one pair of mucosal and full thickness samples was taken from the surgical resection margin (blue arrow) and one or more pairs of samples from within short or longer strictured areas (red arrows). Scale bar 5 cm. (B) Representative ileal sections stained with Masson trichrome showing collagen in blue. Scale bar 1 mm.

Supplementary Table 1. Demographic and clinical characteristics of patient cohort

	**Non-IBD control patients** (n= 11)	**CD stricture patients** (n= 25)	**p value**
**Age at surgery**			
Median (range)*	66 (52-82)	40 (14-80)	0.0007
**Age < 18 years,** n (%)**	0 (0)	3 (12.0)	0.538
**Male**, n (%)**	7 (63.6)	12 (48.0)	0.481
**Medications**, n (%)**			
Anti-TNFα	0 (0)	3 (12.0)	0.538
Corticosteroids	0 (0)	3 (12.0)	0.538
Azathioprine/methotrexate	0 (0)	4 (16.0)	0.290
Ustekinumab	0 (0)	1 (4.0)	> 0.999
Other†	5 (45.5)	1 (4.0)	0.0062
**Surgery type,** n (%)**			
Ileal/ileo-colic resection	0 (0)	25 (100)	< 0.0001
Ileostomy reversal	6 (54.6)	0 (0)	0.0002
Ileostomy formation (anterior resection)	5 (45.5)	0 (0)	0.0012

* Data analyzed by unpaired t test, ** data analyzed by Fisher's exact test, † other medications were beta-blockers, statins and/or proton-pump inhibitors.
